# Effect of repaglinide on endothelial dysfunction during a glucose tolerance test in subjects with impaired glucose tolerance

**DOI:** 10.1186/1475-2840-5-9

**Published:** 2006-04-10

**Authors:** Isabella Schmoelzer, Thomas C Wascher

**Affiliations:** 1 Department of Internal Medicine, Metabolism and Vascular Biology Unit, Medical University of Graz, Auenbruggerplatz 15, 8036 Graz, Austria

## Abstract

**Background:**

Impaired glucose tolerance (IGT) is associated with increased cardiovascular risk. The pathophysiological mechanisms linking post-challenge hyperglycemia to accelerated atherosclerosis, however remain to be elucidated.

**Methods:**

A prospective, open, randomised, cross-over study was performed to investigate the effect of 2 mg repaglinide on hyperglycemia and endothelial function during an oral glucose tolerance test (75 g glucose) in 12 subjects with diagnosed IGT. Blood samples for determination of plasma glucose were drawn fasting, 1 and 2 hours after glucose ingestion. Endothelial function was assessed by measuring flow-mediated dilatation (FMD) of the brachial artery with high-resolution ultrasound.

**Results:**

Administration of repaglinide resulted in a significant reduction of plasma glucose at 2 hours (172.8+/-48.4 vs. 138.3+/-41.2 mg/dl; p < 0.001). The flow-mediated dilatation (FMD) 2 hours after the glucose-load was significantly reduced in comparison to fasting in the control group (6.21+/-2.69 vs. 7.98+/-2.24 %; p = 0.028), whereas after theadministration of repaglinide the FMD was not significantly different to fasting values (7.24+/-2.57 vs. 8.18+/-2.93 %; p = n.s.). Linear and logistic regression analysis revealed that only the change of glucose was significantly correlated to the change of FMD observed (p < 0.001). Regression analysis after grouping for treatment and time confirmed the strong negative association of the changes of plasma glucose and FMD and indicate that the effect of repaglinide observed is based on the reduction glycemia.

**Conclusion:**

In subjects with IGT, the endothelial dysfunction observed after a glucose challenge is related to the extent of hyperglycemia. Reduction of hyperglycemia by repaglinide reduces endothelial dysfunction in a glucose dependent manner.

## Background

Hyperglycemia, across its entire range including non-diabetic values is established as a strong and independent risk factor for cardiovascular morbidity and mortality[1]. In addition, the DECODE study recently established that acute post-challenge glucose concentration, as measured during a standardized glucose tolerance test (oGTT), is a better predictor of risk than fasting glucose also in the non-diabetic range[2]. The mechanisms that link post-challenge hyperglycemia to accelerated atherosclerosis, however, remain to be further elucidated.

Endothelial dysfunction (ED) represents a very early step in the development of atherosclerosis[3]. The reduced nitric-oxide mediated, endothelium-dependent vasodilatation occurring in ED is a predictor of cardiovascular risk in high risk subjects[4] and its improvement seems to predict treatment-induced risk reduction. In diabetic subjects ED already occurs in the fasting state and is aggravated for hours postprandially or in the setting of a glucose-challenge[5]. Patients with impaired glucose tolerance (IGT) on the other hand, most commonly have a normal endothelial function in the fasting state and transient ED during acute hyperglycemia following a glucose-challenge[6]. Although several experimental studies suggest that acute changes of glycemia might lead to endothelial dysfunction[7], it remains to be investigated whether the ED observed following a carbohydrate load is caused by or only associated to hyperglycemia.

Consequently, the aim of our study was to investigate, whether reduction of post-challenge glucose-excursions with repaglinide, a short-acting non-sulfonylurea insulin secretagogue influences endothelial function during an oGTT in patients with IGT.

## Methods

A prospective, randomised, observer-blinded, cross over study was performed to investigate the effect of 2 mg repaglinide on hyperglycemia and endothelial function during an oGTT (75 g glucose dissolved in 300 ml water). 12 subjects (10 males, age 62 ± 4 years, BMI 29.4 ± 5.2 kg/m^2^, waist/hip ratio 0.93 ± 0.03) with IGT according to the WHO-criteria and free of clinically manifest vascular disease were recruited in the study. BMI had to be below 35 kg/m^2^. Blood pressure had to be below 160/90 mmHg at the occasion of investigation. Eventually existing antihypertensive and lipid-lowering medication had to be stable for at least 8 weeks prior and during the study period. On the examination days patient did not take their concomitant medication and smoking was not allowed within 12 hours before the investigations. The study was approved by the local ethics committee and conducted according to the declaration of Helsinki. Signed informed consent was obtained from all subjects prior to study entry.

Subjects were scheduled to undergo investigations at two different occasions, separated by 7 ± 2 days, with or without administration of 2 mg repaglinide at the beginning of the oGTT. Blood sampling and examination of vascular function was performed in the fasting state and repeated 1 and 2 hours after the glucose load. Endothelial function was assessed by measuring flow-mediated dilatation (FMD) of the brachial artery by high-resolution ultrasound as established[8,9] and according to recent local guidelines[10]. Briefly the brachial artery was scanned longitudinally with a linear transducer (7.5 MHz, Acuson Sequoia, Siemens GmbH, Austria) proximal to the antecubital fossa. Depth and gain settings were optimised at the beginning of the examination and the optimal position of the transducer was marked on the skin. All measurements were performed R-wave triggered at the end of diastole. The mean of 3–4 measurements from a stored image was used as baseline diameter. FMD was induced by suprasystolic (>250 mmHg) inflation of a blood pressure cuff, positioned at the forearm, for 5 minutes. Post-ischemic diameter was determined 45–60 seconds after deflation of the cuff at the same position as baseline again from 3–4 measurements and FMD (in%) was calculated (100-(post-ischemic diameter*100/baseline diameter)). At the occasion of the third measurement 2 hours after glucose-challenge also endothelium-independent vasodilatation (NMD) was assessed 4 minutes after administration of 0,4 mg nitroglycerin sublingually. Statistical analysis was performed on an Apple Macintosh using the JMP statistic package (version 5, SAS, Heidelberg, Germany). Comparisons were performed using a paired Student's t-test and a p < 0.05 was considered statistically significant. Linear and logistic regression analysis was performed to investigate associations between glycemia and endothelial function.

## Results

**Figure 1:**
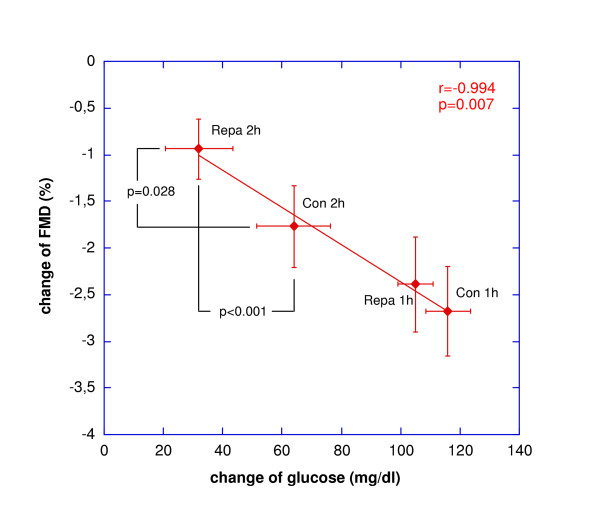
**Glucose and FMD **Association between the change of glucose as well as the change of FMD in the study population grouped according to time and treatment (error bars represent standard deviation)

As to be expected, pronounced hyperglycemia was observed during the oGTT. Administration of repaglinide resulted in a reduction of plasma glucose at 2 hours during the oGTT, accompanied by increased plasma insulin concentration (Tab.1) whereas no effect of the drug could be observed 1 hour after beginning of the test. Flow mediated dilation decreased significantly after 1 hour in both groups. After 2 hours no significant difference from fasting baseline was observed when repaglinide was administered and borderline significance was observed in comparison to the control experiments. In the control group, however, FMD was still significantly reduced at 2 hours. Linear and logistic regression analysis indicated that only the change of glucose was significantly correlated to the change of FMD observed (r: -0.566, p < 0.001). No influence of insulin levels, lipids, age, antihypertensive- or lipid-lowering treatment was detected (data not shown). Regression analysis after grouping for treatment and time (Fig. 1) confirmed the strong negative association of the changes of plasma glucose and FMD respectively and indicated that the beneficial effect of repaglinide observed after 2 hours is based on the reduction of glycemia. No effect of repaglinide in comparison to control was observed with regard to nitroglycerin-mediated, endothelium-independent vasodilatation (Tab. 1).

**Table 1:** Characteristics of study population including glucose values during the OGTT (without repaglinide)

Gender (male/female)	10/2
Age (years)	62 ± 4
BMI (kg/m^2^)	29.4 ± 5.2
Waist/hip ratio	0.92 ± 0.03
Blood pressure (mmHg)	132 ± 12/79 ± 8
Total cholesterol (mg/dl)	220 ± 52
LDL cholesterol (mg/dl)	136 ± 45
HDL cholesterol (mg/dl)	52 ± 12
Triglycerides (mg/dl)	253 ± 126
Fasting glucose (mg/dl)	109 ± 16
1 hour glucose (mg/dl)	214 ± 30
2 hour glucose (mg/dl)	173 ± 48

## Discussion

Experimental studies, supporting the epidemiological evidence, established that acute hyperglycemia induces several additional proatherogenic mechanisms beside ED such as haemostatic alterations, platelet activation, increases of C-reactive protein, expression of adhesion molecules (for review see 11), as well as activation of NFkb[7].

It has been established before that acute hyperglycemia induced by carbohydrate intake is able to induce transient ED in normal subjects and even more pronounced in subjects with IGT[6]. Our study provides evidence that this ED in subjects with IGT is caused by acute postchallenge hyperglycemia and can be prevented by reduction of hyperglycemia. Thus our results extend and confirm those of Ceriello et al. who provided such evidence in subjects with manifest diabetes using subcutaneous insulin as therapeutic strategy[12]. Further recent evidence for the contribution of postprandial glucose to ED comes from a recent study comparing repaglinide and glibenclamide in dietary treated diabetic subjects[13] showing that only repaglinide was able to reduce postprandial glycemia, markers of oxidative stress as well as ED. Insulin itself has been described to induce vasodilatation acutely as well as under chronic conditions. We do, however, not expect that the effect observed during repaglinide treatment is based on an opposing effect of higher insulin levels ameliorating the deleterious effect of hyperglycemia since regression analysis did not exhibit an association between changes of insulin and FMD during the oGTT whereas a highly significant correlation was observed between changes of glucose and FMD.

Our study, however, has several limitations. First, the sample size was rather small and the study was performed in an open design. Second, we observed a somewhat unexpected weak antihyperglycemic effect of repaglinide. We suggest this to be due to the fact that an oGTT instead of a mixed meal was used to induce hyperglycemia. Glucose absorption under these conditions is much faster and was not paralleled by increased insulin release induced by repaglinide. It can be speculated that the effect of repaglinide would have been more pronounced using a mixed meal. Under such conditions, however, additional effects of hyperlipidemia on ED[14] could not have been excluded. Finally given the strong correlation between postchallenge hyperglycemia and postprandial hyperglycemia[15] we suggest the applicability of our results to daily life conditions of subjects with IGT.

## Conclusion

In subjects with IGT, endothelial dysfunction observed after a glucose challenge is related to the extent of hyperglycemia. Reduction of hyperglycemia by repaglinide ameliorates endothelial dysfunction in a glucose dependent manner. These results together with the existing evidence, in our opinion, justify long term investigations into prevention of vascular disease in subjects with IGT by reduction of postprandial hyperglycemia.

## Competing interests

TC Wascher has received several research grants from NovoNordisk and received honoraria as a speaker in scientific symposia organised by NovoNordisk.

## Authors' contributions

IS participated in the design and coordination of the study, carried out the measurements of endothelial function and drafted the manuscript. TCW conceived of the study, participated in its design and coordination, performed the statistical analysis and helped to draft the manuscript. All authors read and approved the final manuscript.

**Table 2:** Plasma insulin and glucose levels as well as FMD during the oGTT (comparisons by paired Student's test, data represent mean ± standard deviation, ^#^median)

	repaglinide	control	p
Fasting Insulin (mU/l)	7.16 ± 4.79	13.06 ± 20.97	n.s.
1 hour Insulin (mU/l)	72.40 ± 62.35	58.32 ± 48.39	n.s.*
2 hour Insulin (mU/l)	120.71 ± 86.78	49.83 ± 36.09	0.013*
Fasting glucose (mg/dl)	106.58 ± 12.38	109.1 ± 16.3	n.s.
1 hour glucose (mg/dl)	222.08 ± 30.05	213.7 ± 29.9	n.s.*
2 hour glucose (mg/dl)	138.33 ± 41.19	172.8 ± 48.4	<0.001***
Fasting FMD (%)	8.18 ± 2.93	7.98 ± 2.24	n.s.
1 hour FMD (%)	5.50 ± 3.11	5.58 ± 2.91	n.s.*
2 hour FMD (%)	7.24 ± 2.57	6.21 ± 2.69	n.s. (0.102)**
1 hour change FMD (%)^#^	-2.25	-1.95	n.s.*
2 hour change FMD (%)^#^	-1.05	-1.6	n.s.*
Fasting NMD (%)	15.09 ± 3.76	15.58 ± 3.74	n.s.

*p < 0.001 vs. fasting for both groups

**p = 0.028 vs. fasting for control, n.s. for repaglinide

***p < 0.001 vs. fasting for control, p = 0.005 for repaglinide
